# The Impact of Transjugular Intrahepatic Portosystemic Shunt on Nutrition in Liver Cirrhosis Patients: A Systematic Review

**DOI:** 10.3390/nu15071617

**Published:** 2023-03-27

**Authors:** Jakub Gazda, Simone Di Cola, Lucia Lapenna, Saniya Khan, Manuela Merli

**Affiliations:** 1 2nd Department of Internal Medicine, Pavol Jozef Safarik University and Louis Pasteur University Hospital, 040 12 Kosice, Slovakia; 2 Department of Translational and Precision Medicine, Sapienza University of Rome, 00185 Rome, Italy

**Keywords:** liver cirrhosis, transjugular intrahepatic portosystemic shunt, nutritional status, sarcopenia, fat tissue

## Abstract

Background and Aims: Liver cirrhosis leads to clinically significant portal hypertension. Transjugular intrahepatic portosystemic shunt (TIPS) has been shown to effectively reduce the degree of portal hypertension and treat its complications. However, poor nutritional status has been shown to be associated with hepatic encephalopathy, acute on chronic liver failure, and mortality following TIPS placement. The purpose of this systematic review is to create another perspective and evaluate the effect of TIPS placement on the nutritional status of patients with liver cirrhosis. Methods: A comprehensive search of four major electronic databases was conducted to identify studies that assessed the nutritional status of cirrhotic patients before and after TIPS placement. The risk of bias was evaluated using ROBINS-I guidelines. Results: Fifteen studies were analyzed in this review. The results indicate that among the 11 studies that evaluated changes in ascites-free weight and body mass index or body cell mass, 10 reported an improvement in one or more measures. Furthermore, all seven studies that evaluated changes in muscle mass demonstrated an increase in muscle mass. Among the four studies that evaluated subcutaneous fat tissue, three showed a significant expansion, while two out of three studies evaluating visceral fat tissue reported a significant reduction. Conclusions: The results of this systematic review suggest that TIPS placement is associated with improvement in the nutritional status of cirrhotic patients, indicated by an increase in ascites-free weight, body mass index, and muscle mass. Additionally, TIPS placement leads to a shift in the distribution of fat mass, with a preference for subcutaneous over visceral adipose tissue. Notably, sarcopenic patients seem to benefit the most from TIPS placement in terms of nutritional status.

## 1. Introduction

Liver cirrhosis leads to clinically significant portal hypertension (CSPH) in 40–70% of patients, increasing the risk of decompensation and death [1,2]. TIPS (transjugular intrahepatic portosystemic shunt) has been shown to effectively reduce the degree of portal hypertension and treat complications such as recurrent/refractory ascites and bleeding from gastroesophageal varices [2]. Cirrhosis and portal hypertension often result in malnutrition and sarcopenia, due to factors such as reduced caloric intake and increased catabolism [3,4]. Eighty percent of decompensated cirrhosis patients experience sarcopenia that further worsens with liver decompensation [5]. These conditions raise the risk of increased mortality and decompensation [6,7]. Interestingly, even without impaired liver function, portal hypertension alone may increase the risk of sarcopenia [8]. Given this relationship, TIPS placement often occurs in the context of malnutrition. Sarcopenia has been shown to be associated with hepatic encephalopathy (HE), acute-on-chronic liver failure (ACLF), and mortality following TIPS placement [9,10,11,12,13]. As a result, sarcopenia is now considered a relative contraindication to TIPS placement in current guidelines [2]. On the other hand, recent evidence suggests that TIPS placement may improve the nutritional status of cirrhotic patients by increasing the fat-free muscle mass and reducing visceral fat [10,14,15]. However, the optimal timing for TIPS placement in sarcopenic cirrhotic patients remains unknown. The purpose of this systematic review is to examine the current literature and determine the true impact of TIPS placement on the nutritional status of cirrhotic patients.

## 2. Materials and Methods

The PICO framework was followed to define the review question’s key elements [16]. The reporting of this systematic review and meta-analysis follows the Preferred Reporting Items for Systematic Reviews and Meta-Analyses (PRISMA) statement (http://www.prisma-statement.org (accessed on 14 December 2022)) [17].

### 2.1. Literature Search

The MEDLINE (PubMed), Scopus, Web of Science, and Cochrane library databases were comprehensively searched from inception to 14 December 2022. A literature review was created using the following terms (with corresponding variations): ((cirrhosis (MeSH Terms) OR (ascites) OR (hepatic encephalopathy) OR (cirrhotics)) AND (Portosystemic Shunt, Transjugular Intrahepatic (MeSH Terms)) AND ((Nutritional Status (MeSH Terms)) OR (nutritional) OR (albumin) OR (BMI) OR (total body mass) OR sarcopenia OR (body composition) OR (body weight))). Medical Subject Headings (MeSH) were used to increase the precision and efficiency of the search. Additionally, we manually checked the reference lists of the included studies (or relevant review articles) and performed a backward citation analysis.

### 2.2. Criteria for the Selection of Studies

Inclusion criteria for the studies consisted of evaluations of patients diagnosed with liver cirrhosis who underwent transjugular intrahepatic portosystemic shunt (TIPS) placement, regardless of the indication for the procedure. Additionally, the studies were required to assess changes in nutritional status of the patients prior to and following TIPS insertion. The exclusion criteria for the studies consisted of any abstracts, studies that utilized animal models, case reports, and correspondence letters. Additionally, the scope of the literature search was restricted to articles written in the English, German, Italian, and Spanish languages.

### 2.3. Study Selection, Data Extraction, and Data Calculation

A standardized data extraction sheet was developed, and subsequently underwent pilot testing to ensure its reliability. Two investigators independently performed a literature review, utilizing titles and abstracts as initial screening criteria (JG, SK). Eligible full-text articles were subsequently assessed, and relevant data were extracted by the investigators (JG, SK). The extracted information was then reciprocally compared between the investigators (JG, LP, SD, SK). Any discrepancies in study selection or data extraction were resolved through a consensus process, with the assistance of a senior hepatologist (MM).

The following pre-determined data points were extracted from each eligible study: (1) First author’s name, (2) Year of publication, (3) Study design, (4) Time frame of the study, (5) Patient exclusion criteria, (6) Sample size, (7) Characteristics of the study participants, (8) Indication for TIPS procedure, (9) Characteristics of the TIPS procedure, (10) Portal pressure gradient post-TIPS procedure, (11) Follow-up duration, (12) Measures of evaluating nutritional status, and (13) Change in nutritional status. In instances where the authors did not directly report the change in nutritional status, it was calculated from the available information, if feasible.

### 2.4. Risk of Bias Assessment

The risk of bias is evaluated using the ROBINS-I guidelines, which identify seven domains and classify the risk as low, moderate, serious, or critical. A low risk of bias indicates that the study is similar to a well-conducted randomized trial, while a critical risk indicates significant problems with the study [18].

## 3. Results

**Figure 1:**
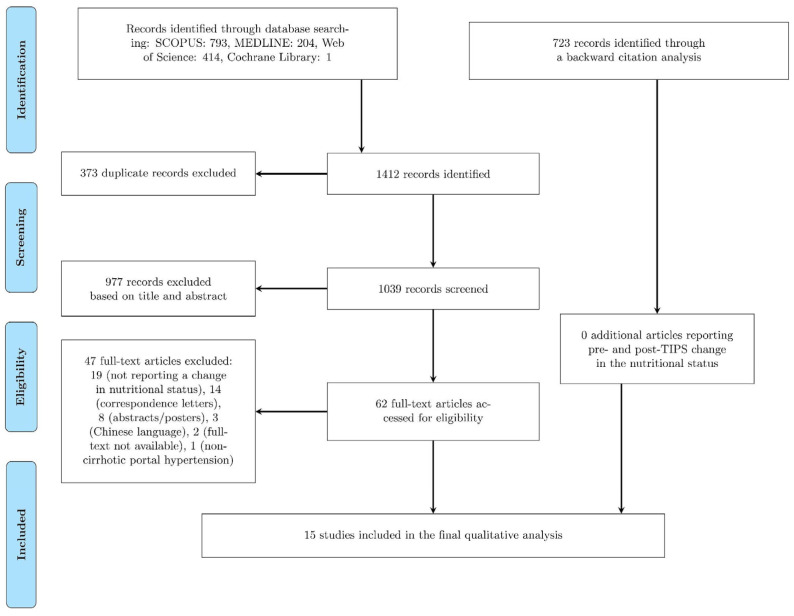
Flow of information through different phases of the systematic review.

A comprehensive search of four major medical databases (MEDLINE, Scopus, Web of Science, and Cochrane) yielded a total of 1412 records. A total of 373 duplicate records were subsequently removed. Of the remaining 1039 records, 62 were evaluated in full-text, and 47 were excluded based on pre-determined exclusion criteria (as detailed in Figure 1). Additionally, 723 additional records were identified through backward citation analysis. In total, 15 records were finally included in the present study (as illustrated in Figure 1).

In the present study, a total of 15 studies were included (Table 1, more detailed in Supplementary Table S1), which were published between 1998 and 2022, and altogether followed 850 patients. Of these, seven studies (47%) were retrospective in design [13,14,19,20,21,22,23], three studies (20%) were prospective [24,25,26], and five studies (33%) did not disclose this information [15,27,28,29,30]. In the majority of the studies, the sample population consisted of all consecutive patients, with exclusion criteria based on cardiac, renal, hepatic, or pulmonary function, as well as a history of malignancy. The most frequent underlying chronic liver disease was alcohol-related liver disease (in 10 out of 15 included studies; 67%). The sample sizes of the included studies varied between 11 and 224 patients, and the mean or median age of the patients varied between 54.1 and 60 years, with male patients predominating in all studies. The indications for TIPS insertion were refractory ascites and variceal bleeding. The most frequently used stent material was polytetrafluoroethylene (PTFE); however, some older studies employed bare metallic stents. The proportion of patients with TIPS dysfunction was reported infrequently [19,20,26]. In instances of dysfunction detection, intervention in the form of TIPS revision was implemented to re-establish patency. Non-responsive cases were excluded from the original studies. The post-TIPS portal pressure gradient varied between studies, ranging from 6.0 to 15.5 mmHg. Furthermore, the time point at which the change in nutritional status was measured varied between 2 and 36 months after TIPS insertion.

### 3.1. Risk of Bias Assessment

**Figure 2:**
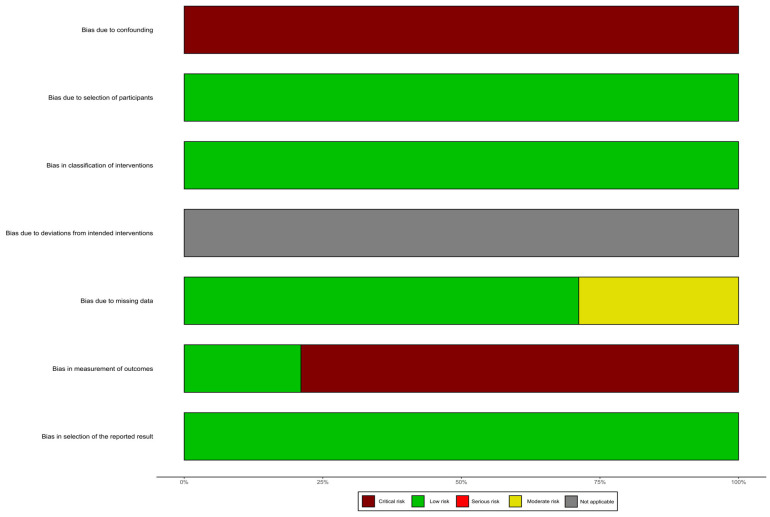
Risk of bias assessment according to the ROBINS-I guidelines (Risk of Bias in Non-randomized Studies of Interventions).

In this systematic review, we did not assess the risk of bias related to deviations from the intended interventions. We found a critical risk of bias due to confounding, as none of the studies controlled for variables such as caloric intake or the frequency and intensity of physical exercise. On the other hand, the risk of bias due to participant selection was low, as all eligible and consecutive patients were included. The risk of bias related to the classification of interventions was also low. The risk of bias due to missing data was moderate in some studies, as a number of patients were lost to follow-up. However, the risk of bias related to the measurement of outcomes was critical, as most evaluators of nutritional status were not blinded to the intervention. Lastly, we did not detect any evidence of selective reporting of results (Figure 2).

The subsequent section of the results is divided into three distinct sub-sections. The first sub-section presents the changes in Body Mass Index (BMI), weight (W), and/or body cell mass (BCM). The second and third sub-sections present the changes in body composition with regard to alterations in muscle and fat tissue, respectively.

### 3.2. Body Mass Index, Weight, and/or Body Cell Mass

In total, 11 studies reported on changes in Body Mass Index (BMI, kg/m^2^), weight (W, kg), and/or body cell mass (BCM, kg) [15,20,22,23,24,25,26,27,28,29,30] (Table 1, more detailed in Supplementary Table S1). These measures were evaluated in patients with no ascites (following peritoneal paracentesis) or were adjusted for ascites volume. Of these studies, six reported a significant improvement in BMI or W [20,22,23,24,25,27], with two studies noting improvement in BCM only (the increase in BMI/weight was in these cases statistically not significant) [28,30]. Two studies that specifically distinguished between underweight or sarcopenic patients and those who were overweight or nonsarcopenic reported a significant improvement in nutritional status in the former group [20,26]. Finally, only one study failed to report an improvement in such measures [15].

### 3.3. Skeletal Muscle Volume, Skeletal Muscle Function

A total of seven studies reported on changes in skeletal muscle volume [13,14,15,19,20,21,24] and skeletal muscle function [27] (Table 1, more detailed in Supplementary Table S1). The mid-arm muscle area (MAMA) was quantitatively determined using a flexible tape measure [24]. To further assess muscle mass, axial computed tomography (CT) images were obtained at an L3 vertebra height through the abdomen and utilized to calculate skeletal muscle areas (SMA) [15,20,21]. These muscle areas were then normalized to height, resulting in the calculation of the skeletal muscle index (SMI) [13,14,20]. Additionally, both transversal right psoas muscle thickness at the umbilical level/height (TPMT/height in mm/m) and total psoas muscle area (TPMA in mm^2^) were also reported in the study by Artru et al. [19]. All studies measuring changes in skeletal muscle volume reported a significant improvement, with Liu et al. confirming this trend specifically in sarcopenic patients [20]. The SMA increased by a minimum of 6.6 cm^2^ within 12 months after TIPS placement [15,21]. This increase was even more pronounced in sarcopenic patients, with an increase of up to 20 cm^2^ [20]. The improvement in SMI ranged between 2.39 and 5.8 cm^2^/m^2^, with sarcopenic patients showing an even greater improvement of approximately 8 cm^2^/m^2^ within 10–19 months after TIPS placement [13,14,20]. Additionally, both the TPMT and TPMA significantly improved within 6 months of TIPS placement [19]. On the other hand, the study conducted by Allard et al. found that there was no improvement in measures of skeletal muscle function, such as muscle relaxation rate and muscle force [27].

### 3.4. Adipose Tissue Volume

In a total of nine studies, changes in the quantity of adipose tissue were evaluated [14,15,19,20,24,26,27,29,30] (Table 1, more detailed in Supplementary Table S1). To assess adipose tissue quantity, axial computed tomography (CT) images were obtained at an L3 height through the abdomen. The volume of subcutaneous adipose tissue (SAT, adipose tissue below the skin but above the parietal peritoneal lining in cm^3^/3 mm) and visceral adipose tissue (VAT, intraperitoneal adipose tissue in cm^3^/3 mm) were estimated [15]. Furthermore, adipose tissue areas were normalized to height, resulting in the estimation of the tissue area indices—subcutaneous adipose tissue index and visceral adipose tissue index (SATI and VATI in cm^2^/m^2^) [14]. Subcutaneous and visceral fat surfaces (SFA and VFA in cm^2^, respectively) were also calculated [19,20]. Additionally, Liu et al. quantified subcutaneous fat thickness (SFT in mm) [20]. Fat mass (% of body weight or in kg) was estimated using calorimetry [26,27,29,30]. Finally, Plauth et al. quantified the mid-arm fat area using a skinfold caliper (MAFA in cm^2^) [24]. In the reviewed studies, no significant change was observed in measures of fat mass (measured using calorimetry, except an increase in the study by Allard et al.) or in the mid-arm fat area [24,26,27,29,30]. However, a significant expansion of subcutaneous fat tissue was observed in all studies [14,19,20], with the exception of one study [15]. Additionally, a notable reduction in visceral fat tissue was reported in two studies [14,19], while remaining unchanged in one study [15].

**Table 1:** Main changes in the nutritional status after transjugular intrahepatic portosystemic shunt placement in cirrhotic patients.

Reference	Design	Sample Size	TIPS Indication	Follow-Up after	Measure	Change
Allard et al. (2001) [27]	-	14 (71% ♂)	RA (100%)	12 M	W, Dry W, FM, F10/F30, and MRR	Significant increase in Dry W and FM.
Artru et al. (2020) [19]	RS	179 (72% ♂)	RA (47.5%), VB (52.5%)	6 M	TPMT, TPMA, SFA, and VFA	Significant increase in TPMT, TPMA, and SFA and significant decrease in VFA.
Gioia et al. (2019) [13]	RS	27 (85% ♂)	RA (56%), VB (44%)	9.8 M	SMI	Significant increase in SMI.
Gioia et al. (2021) [14]	RS	35 (80% ♂)	RA (54%), VB (46%)	19 M	SMI, SATI, and VATI	Significant increase in SMI and SATI and significant decrease in VATI.
Holland-Fischer et al. (2010) [29]	-	11 (73% ♂)	RA (64%), RA+VB (36%)	6 M	W, BMI, BCM, LBM, and FM	Significant increase in all but FM.
Holland-Fischer et al. (2009) [28]	-	17	RA (59%), VB (29%), both (12%)	13 M	W and BCM	Significant increase in BCM.
Jahangiri et al. (2019) [21]	RS	76 (56.2% ♂)	RA/RH(52.6%), VB (47.4%)	13.5 M	SMA	Significant increase in SMA.
Liu et al. (2022) [20]	RS	224 (71% ♂)	RA (14%), VB (86%)	12 M	SMA, SMI, SFA, SFT, AF W, and AF BMI	No significant change in SMA, SMI, SFA, and SFT in patients without sarcopenia. Significant increase in SMA, SMI, SFA, and SFT in patients with sarcopenia. No significant change in AF W and AF BMI in patients without ascites and sarcopenia. Significant increase in AF W and AF BMI in patients with sarcopenia but without ascites.
Montomoli et al. (2010) [26]	PS	21	RA (57%), VB (33%), both (10%)	13 M	BMI, FM, and DLM	No significant change in overweight patients, significant increase in dry lean mass in under/normal weight patients.
Nolte et al. (2003) [25]	PS	31	RA, VB	9 M	W, BMI, AF W, and AF BMI	Significant increase in W, BMI, AF W, and AF BMI in male patients, significant increase in AF W and AF BMI in female patients.
Pang et al. (2021) [22]	RS	77	RA, VB	13 M	W, BMI	Significant increase in W and BMI.
Plauth et al. (2004) [24]	PS	21 (62% ♂)	RA (33%), VB (43%), both (24%)	12 M	W, BMI, MAFA, MAMA, and BCM	Significant increase in W, BMI, and MAMA.
Thomsen et al. (2012) [30]	-	25 (60% ♂)	RA (68%), VB (20%), both (12%)	6 M	W, BMI, FM*, and BCM	Significant increase in BCM.
Trotter et al. (1998) [23]	RS	35 (69% ♂)	RA	8.8 M	W	Significant increase in W.
Tsien et al. (2012) [15]	-	57 (63% ♂)	RA (72%), VB (25%), both (3%)	13.5 M	BMI, SMA, VAT, and SAT	Significant increase in SMA and significant decrease in SAT.

Footnote: AF—ascitic-free, ASC—ascites, BCM—body cell mass (kg), BMI—body mass index (kg/m^2^), DLM—dry lean mass (kg), FM—fat mass (% of total body weight/*kg), F10/F30—force of m. adductor policis (%), kg—kilogram, M—months, MAFA—mid-arm fat area, MAMA—mid-arm muscle area (cm^2^), MRR—muscle relaxation rate (m. adductor policis) (%), SAT—subcutaneous adipose tissue (cm^3^/3 mm), SATI—subcutaneous adipose tissue index (cm^2^/m^2^), SFA—subcutaneous fat area (cm^2^), SFT—subcutaneous fat thickness (cm), SMA—skeletal muscle area (cm^2^), SMI—skeletal muscle index (cm^2^/m^2^), TPMA—total psoas muscle area (mm^2^), TPMT—transversal right psoas muscle thickness at the umbilical level/height (mm/m), TIPS—transjugular intrahepatic portosystemic shunt, VAT—visceral adipose tissue (cm^3^/3 mm), VATI—visceral adipose tissue index (cm^2^/m^2^), VFA—visceral fat area (cm^2^), W—weight (kg), -—no information. A more detailed table is provided within the Supplementary Materials.

## 4. Discussion

TIPS placement is a commonly utilized therapeutic intervention for individuals with liver cirrhosis and associated complications of portal hypertension, including refractory ascites and variceal bleeding. This intervention has been demonstrated to significantly improve overall survival in these patients, as evidenced by several clinical studies [31,32]. Despite these benefits, malnutrition, which is prevalent among cirrhotic individuals, has been linked to adverse outcomes after TIPS placement, such as hepatic encephalopathy, acute-on-chronic liver failure (ACLF), and mortality [10,11,12]. On the other hand, some studies have suggested an improvement in the nutritional status following TIPS placement in cirrhotic patients. Therefore, the aim of this systematic review was to evaluate changes in nutritional status that occur after TIPS placement in patients with liver cirrhosis.

This review analyzed data from 15 studies (comprising a total of 850 patients) that were published between 1998 and 2022. The number of participants per study ranged from 11 to 224, with the majority being men. Most of the participants in these studies had alcohol-related liver disease. Alcohol consumption has a multifactorial and complex impact on nutritional status. Firstly, heavy alcohol consumption can significantly reduce dietary intake. Secondly, the metabolism of ethanol involves energy-wasting pathways. Thirdly, chronic alcohol consumption can result in the wastage of fat and muscle. Fourth, many alcohol-related diseases can interfere with dietary intake and contribute to malnutrition, such as chronic alcoholic gastritis, chronic pancreatitis, and chronic liver disease [33]. Finally, alcohol decreases muscle protein synthesis via inhibition of mTOR-dependent translation initiation [34]. All studies included in this systematic review considered active alcohol abuse as an exclusion criterium for patient enrollment; therefore, we should not consider alcohol consumption as an active player in the nutritional modifications reported in these studies.

The indications for TIPS insertion were refractory ascites and variceal bleeding, the most commonly used stent material in the studies was polytetrafluoroethylene, and the post-TIPS portal pressure gradients ranged from 6.0 to 15.5 mmHg. Although the patient populations might have overlapped in some studies [26,28,29,30], we decided to include these studies because they provided unique information, such as a specific measure of nutritional status or a different time point since TIPS insertion.

The results of the qualitative analysis showed marked improvements in muscle mass and a shift in fat tissue distribution after TIPS placement. There are several potential explanations for the observed changes in body composition after TIPS placement. These changes may be related to the reduction in portal hypertension [8] and its associated effects on gut permeability, bacterial translocation, proinflammatory cytokines, and chronic inflammation [35,36,37,38]. Additionally, the TIPS procedure may improve protein-losing enteropathy and reduce frequent hospitalizations due to gastrointestinal bleeding and paracentesis, leading to improved mobility and oral intake. Unfortunately, only limited information was available regarding dietary changes after TIPS. In a study by Tsien et al., only 25% of patients reported an increase in their total dietary intake [15].

It is worth noting the relationship between the nutritional status before TIPS and the reported nutritional improvement after the procedure. Two studies found that the improvement in nutritional status was more pronounced in patients with sarcopenia or who were underweight or of normal weight, rather than in overweight or non-sarcopenic individuals [20,26]. Liu et al. found an increase in SMA, SMI, SFA, and SFT in sarcopenic patients, while they did not observe any significant change in non-sarcopenic patients. Montomoli et al. found an increase in dry lean mass in the group of normal or underweight patients but not in the group of overweight patients. Tsien et al. also reported that younger age, male sex, and lower pre-TIPS muscle area were independent predictors of increased muscle mass after TIPS [15].

These findings create a sort of paradox, as malnutrition and sarcopenia have been associated with increased risk of adverse outcomes after TIPS placement, namely, hepatic encephalopathy, acute on chronic liver failure, or mortality [9,10,11,12,13]. This was the reason why the latest Baveno VII consensus discouraged sarcopenia itself as an indication for TIPS insertion [2], though these patients are likely to benefit the most from TIPS in terms of nutritional improvement. When considering an elective TIPS procedure for a malnourished sarcopenic patient, clinical judgment must account for two factors: the short-term risks of the procedure and the potential long-term benefits—correcting portal hypertension and improving the patient’s nutritional status.

Several studies have also observed alterations in adipose tissue composition following TIPS placement. Significant expansion of subcutaneous fat tissue was observed in all studies [14,19,20], with the exception of one study [15]. Additionally, a notable reduction in visceral fat tissue was reported in two studies [14,19], while remaining unchanged in one study [15]. There is evidence to suggest that the procedure leads to a decrease in VAT and an increase in SAT. The venous drainage of VAT occurs directly via the portal circulation to the liver. Therefore, changes in portal circulation subsequent to TIPS placement may enhance the availability of these fat deposits to be metabolized as energy sources by the liver [39,40]. Additionally, increased circulating levels of adipokines have been observed post-TIPS placement, potentially reflecting anabolic changes that contribute to the alterations in adipose tissue observed in patients undergoing TIPS [30].

The considerable clinical and methodological heterogeneity between studies precluded the conduct of a quantitative analysis (meta-analysis). The variability in nutritional status evaluation across studies was identified as a major contributing factor to this limitation. Indeed, the methods used to evaluate nutritional status varied widely across studies, including the use of Body Mass Index (BMI), weight, body cell mass, skeletal muscle volume and function, and measures of adipose tissue. Furthermore, the time interval between TIPS placement and nutritional reassessment ranged from 1–3 to 19 months. Shorter follow-up times might have limited the observation of significant changes, while longer follow-up times may have resulted in significant selection bias, as patients who had died or undergone transplantation were more likely to be excluded from the analysis.

Finally, it is crucial to conduct well-designed prospective studies using gold-standard methods for evaluating malnutrition, in order to identify patients who would most benefit from TIPS placement, including from a nutritional perspective. These efforts could provide not only new pathophysiological insights into the relationship between portal hypertension and malnutrition, but could also answer the question of the optimal timing for TIPS insertion in sarcopenic cirrhotic patients.

## 5. Conclusions

In conclusion, the results of this review indicate an improvement in ascites-free weight and body mass index following the TIPS procedure. This improvement has been associated with an increase in muscle mass, as evidenced by various measures such as the skeletal muscle index or skeletal muscle area. Additionally, TIPS placement leads to a redistribution of fat mass, with a preference for subcutaneous over visceral adipose tissue. Sarcopenic patients seem to benefit the most from TIPS placement in terms of nutritional status.

## Data Availability

The data used to support these findings are all available within the article (and/or) its Supplementary Material.

## Supplementary Materials

The following supporting information can be downloaded at: https://www.mdpi.com/article/10.3390/nu15071617/s1.
