# Supplementary material for: The Impact of Transjugular Intrahepatic Portosystemic Shunt on Nutrition in Liver Cirrhosis Patients: A Systematic Review
Source: Nutrients. 2023 Mar 27;15(7):1617. doi: 10.3390/nu15071617 (PMC10096634; doi:10.3390/nu15071617)
Supplement: Supplementary file 1 [file nutrients-15-01617-s001.zip › nutrients-2247170-supplementary.pdf]

Supplementary Material to a Review Article Entitled: “The Impact of Transjugular Intrahepatic Portosystemic Shunt on Nutrition in Liver Cirrhosis Patients: A Systematic Review”

Jakub Gazda 1, Simone Di Cola 2, Lucia Lapenna 2, Saniya Khan 2 and Manuela Merli 2,*

1 2nd Department of Internal Medicine, Pavol Jozef Safarik University and Louis Pasteur University Hospital, 040 12 Kosice, Slovakia

2 Department of Translational and Precision Medicine, Sapienza University of Rome, 00185 Rome, Italy

* Correspondence: manuela.merli@uniroma1.it; Tel.: +39-06-49972001

Table S1. Changes in the nutritional status after transjugular intrahepatic portosystemic shunts insertion in cirrhotic patients

Reference	Design	Time	Exclusion criteria	Sample Size	Participant Characteristics	TIPS Indication	TIPS Model	PPG after TIPS	Follow-up after	TIPS dysfunction	Measure	Change
Allard et al. (2001) [27]	-	-	-	14 (71% ♂)	57.5±2.2 years, CP 9	RA (100%)	-	-		-		
				12					3 M		W	-0.1 kg (NSD)
				10					12 M		W	-0.3 kg (NSD)
				12					3 M		Dry W	+6.5 kg (p<0.001)
				10					12 M		Dry W	+7.4 kg (p=0.008)
				12					3 M		FM	-0.67 (NSD)
				10					12 M		FM	+4.39 (p<0.001)
				12					3 M		F10/F30	+1.74 (NSD)
				10					12 M		F10/F30	+5.14 (NSD)
				12					3 M		MRR	-0.25 (NSD)
				10					12 M		MRR	+0.2 (NSD)
Artru et al. (2020) [19]	RS	Jul 2011 – Mar 2017	-	179 (72% ♂)	58.2 years (IQR 11.9), CP 8, MELD 11.4	RA (47.5%), VB (52.5%)	8 and 10mm PTFE-C alone or with BMS	<10mmhg				
				128, 85					1-3, 6M	49 (38%), 31 (37%)	TPMT	+0.6, +2.1 (p=0.004, <0.001)
				128, 85					1-3, 6M	49 (38%), 31 (37%)	TPMA	+98, +244.9 (p<0.001, <0.001)
				128, 85					1-3, 6M	49 (38%), 31 (37%)	SFA	+9.6, +46.4 (p<0.001, <0.001)
				128, 85					1-3, 6M	49 (38%), 31 (37%)	VFA	-23, -22.3 (p<0.001, 0.009)
Gioia et al. (2019) [13]	RS	Jan 2015 – Jan 2016	Inadequate L, K, C or P functions, >75years, HCC, infection, SPB, PVT	27 (85% ♂)	58.07±6.7 years, CP 7.1, MELD 11.3	RA (56%), VB (44%)	10mm PTFE-C	-		-		
				27					9.8 M		SMI	+5.8 (p<0.001)
				27					9.8 M		MA	+3.2 (p=0.006)
Gioia et al. (2021) [14]	RS	Jan 2017 – Dec 2020	Inadequate L, K, C or P functions, >75years, HCC, infection, SPB, PVT	35 (80% ♂)	58.6±6.3 years, CP 7.9, MELD 11.4	RA (54%), VB (46%)	10mm PTFE-C	-		-		
									19M		SMI	+2.39 (p=0.04)
									19M		MA	+3.68 (p=0.003)
									19M		SATI	+15.9 (p=0.004)
									19M		VATI	-9.2 (p=0.007)
Holland-Fischer et al. (2010) [29]	-	-	-	11 (73% ♂)	58±4 years, MELD 10	RA (64%), RA+VB (36%)	PTFE-C	-		-		
				11					6M		W	+6.4 (p<0.001)
				11					6M		BMI	+2.2 (p=0.009)
				11					6M		BCM	+4.8 (p=0.002)
				11					6M		LBM	+5.7 (p=0.001)
				11					6M		FM	-1.1 (NSD)
Holland-Fischer et al. (2009) [28]	-	-	-	17	56 years, MELD 8	RA (59%), VB (29%), both (12%)	-	6mmHg		-		

Table S1. Changes in the nutritional status after transjugular intrahepatic portosystemic shunts insertion in cirrhotic patients

Reference	Design	Time	Exclusion criteria	Sample Size	Participant Characteristics	TIPS Indication	TIPS Model	PPG after TIPS	Follow-up after	TIPS dysfunction	Measure	Change
									13 M		W	+4.3 (NSD)
									13 M		BCM	+3.3 (p<0.05)
Jahangiri et al. (2019) [21]	RS	Apr 2004 – Dec 2015	-	76 (56.2% ♂)	54.2±11.2 years, MELD 16	RA/RH(52.6 %), VB (47.4%)	PTFE-C	-		-		
				76					13.5 M		SMA	+6.6 (p=0.002)
				76					13.5 M		MA psoas	+3.1 (p=0.02)
				76					13.5 M		MA paraspinal	+1.5 (NSD)
				76					13.5 M		MA abdom. wall	+4.7 (NSD)
				13					6,12,18M		SMA	+ (p<0.001, <0.1, <0.1)
Liu et al. (2022) [20]	RS	Aug 2016 – May 2020	Inadequate L or K functions, HCC (or other malignancy), infection after TIPS, CHD, uncontrolled DM or AH	224 (71% ♂)	54.3±11.6 years, CP 7.7, MELD 11.9	RA (14%), VB (86%)	8mm BM and 8 mm PTFE-C inside of the BMS	RA: 9 mmHg, VB: 12mmHg		23 (10%)		
				>48	Nonsarcopenic ♂				2, 5, 12 M		SMA	+0,5, +2.1, +3.2 (NSD, NSD, NSD)
				>48	Nonsarcopenic ♂				2, 5, 12 M		SMI	+0.3, +1.0, +1.5 (NSD, NSD, NSD)
				>48	Nonsarcopenic ♂				2, 5, 12 M		SFA	-0.5, -1.1, +14.7 (NSD, NSD, NSD)
				>48	Nonsarcopenic ♂				2, 5, 12 M		SFT	+0.9, +1.7, +1.9 (NSD, NSD, NSD)
				>48	Nonsarcopenic ♀				2, 5, 12 M		SMA	-1.0, +3.3, +2.8 (NSD, NSD, NSD)
				>48	Nonsarcopenic ♀				2, 5, 12 M		SMI	-0.4, +1.3, +1.1 (NSD, NSD, NSD)
				>48	Nonsarcopenic ♀				2, 5, 12 M		SFA	-6.5, +13.8, +9.3 (NSD, NSD, NSD)
				>48	Nonsarcopenic ♀				2, 5, 12 M		SFT	+0.5, +1.5, +2.4 (NSD, NSD, NSD)
				>95	Sarcopenic ♂				2, 5, 12 M		SMA	+3.6, +21.3, +22.8 (NSD, p<0.001, <0.001)
				>95	Sarcopenic ♀				2, 5, 12 M		SMA	+4.1, +17.7, +21.2 (p=0.03, <0.001, <0.001)
				>95	Sarcopenic ♂				2, 5, 12 M		SMI	+1.3, +7.3, +7.8 (p=0.13, <0.001, <0.001)
				>95	Sarcopenic ♀				2, 5, 12 M		SMI	+1.6, +6.9, +8.3 (p=0.02, <0.001, <0.001)
				>95	Sarcopenic ♂				2, 5, 12 M		SFA	+0.7, +20.9, +24.4 (NSD, p<0.001, <0.001)
				>95	Sarcopenic ♀				2, 5, 12 M		SFA	+1.8, +23.6, +28.2 (NSD, p<0.001, <0.001)
				>95	Sarcopenic ♂				2, 5, 12 M		SFT	+1.0, +3.5, +3.9 (p=0.05, <0.001, <0.001)
				>95	Sarcopenic ♀				2, 5, 12 M		SFT	-0.6, +1.7, +2.7 (NSD, NSD, <0.001)
				30	NoASC NoSarcoP ♂				12 M		AF W	NSD
				18	NoASC NoSarcoP ♀				12 M		AF W	NSD
				30	NoASC NoSarcoP ♂				12 M		AF BMI	NSD
				18	NoASC NoSarcoP ♀				12 M		AF BMI	NSD
				30	NoASC + SarcoP ♂				12 M		AF W	+7 (p=0.008)
				22	NoASC + SarcoP ♀				12 M		AF W	+5.5 (p=0.004)
				30	NoASC + SarcoP ♂				12 M		AF BMI	+2.4 (p=0.01)
				22	NoASC + SarcoP ♀				12 M		AF BMI	+2.2 (p=0.002)
Montomoli et al. (2010) [26]	PS	-	-	21	MELD 11	RA (57%), VB (33%), both (10%)	PTFE-C	6mmHg		3 (14%)		
				21					13M		BMI	+1.2 (NSD)
				21					13M		FM	-1.6 (NSD)

Table S1. Changes in the nutritional status after transjugular intrahepatic portosystemic shunts insertion in cirrhotic patients

Reference	Design	Time	Exclusion criteria	Sample Size	Participant Characteristics	TIPS Indication	TIPS Model	PPG after TIPS	Follow-up after	TIPS dysfunction	Measure	Change
				21					13M		DLM	+0.8 (NSD)
				12	UNW			6.3mmHg	13M		BMI	+2.7 (NSD)
				12	UNW			6.3mmHg	13M		FM	-2.3 (NSD)
				12	UNW			6.3mmHg	13M		DLM	+1.8 (p=0.03)
				9	OW			5.7mmHg	13M		BMI	+1.5 (NSD)
				9	OW			5.7mmHg	13M		FM	-1.0 (NSD)
				9	OW			5.7mmHg	13M		DLM	-0.3 (NSD)
Nolte et al. (2003) [25]	PS	1998-2000	tense ascites, first bleeding episode, non-abstinence	31	CP A - 50-53%, CP B 37-38%, CP C 10-12%	RA, VB	-	-		-		
				19, 15♂					3, 9M		W	+0.6, +2.2 (NSD, p=0.04)
				19, 15♂					3, 9M		BMI	+0.2, +0.7 (NSD, p=0.04)
				19, 15♂					3, 9M		AF W	+2.1, +3.6 (NSD, p=0.02)
				19, 15♂					3, 9M		AF BMI	+0.6, +1.2 (NSD, p=0.02)
				12, 9♀					3, 9M		W	-0.8, +0.9 (NSD, NSD)
				12, 9♀					3, 9M		BMI	-0.3, -0.4 (NSD, NSD)
				12, 9♀					3, 9M		AF W	+2.6, +4.8 (p=0.02, 0.003)
				12, 9♀					3, 9M		AF BMI	+1.1, +2.2 (p=0.004, 0.001)
Pang et al. (2021) [22]	RS	Nov 2017 – Aug 2018	C, K, P functions, HE	77	54.1±12.2 years	RA, VB	PTFE-C	-		-		
									13M		W	+2.1 (p<0.01)
									13M		BMI	+0.8 (p<0.01)
				-					1-6M		W	+ (p<0.05)
				-					7-12M		W	+ (p<0.05)
				-					13-36M		W	+ (p<0.05)
				-					>36M		W	+ (NSD)
				-					1-6M		BMI	+ (p<0.05)
				-					7-12M		BMI	+ (p<0.05)
				-					13-36M		BMI	+ (p<0.05)
				-					>36M		BMI	+ (NSD)
Plauth et al. (2004) [24]	PS	-	-	21 (62% ♂)	60 years	RA (33%), VB (43%), both (24%)	-			-		
				21				15.5mmHg	6M		W	+8 (0.001)
				21				15.5mmHg	6M		BMI	+3.9 (p<0.001)
				21				15.5mmHg	6M		MAMA	+5.4 (p=0.001)
				21				15.5mmHg	6M		MAFA	+2 (NSD)
				21				15.5mmHg	6M		BCM	+4.4 (p<0.025)
				16				11.5mmhg	12M		W	+0.9 (p<0.01)

Table S1. Changes in the nutritional status after transjugular intrahepatic portosystemic shunts insertion in cirrhotic patients

Reference	Design	Time	Exclusion criteria	Sample Size	Participant Characteristics	TIPS Indication	TIPS Model	PPG after TIPS	Follow-up after	TIPS dysfunction	Measure	Change
				16				11.5mmhg	12M		BMI	+ (p<0.025)
				16				11.5mmhg	12M		MAMA	+ (p<0.001)
				16				11.5mmhg	12M		BCM	+ (NSD)
Thomsen et al. (2012) [30]	-	-	-	25 (60% ♂)	53 years, MELD 8.6	RA (68%), VB (20%), both (12%)	-	6mmHg		-		
									6M		W	+5 (NSD)
									6M		BMI	+1 (NSD)
									6M		FM*	-1.8 (NSD)
									6M		BCM	+3.6 (p=0.03)
Trotter et al. (1998) [23]	RS	Nov 1994 – Aug 1997	K and P functions, HE	35 (69% ♂)	54.0±9.2 years, CP B 86%, CP C 14%	RA	10mm	8.1mmHg				
									2M		W	-6.1 (p<0.05)
									8.8M		W	+5.5 (p<0.05)
Tsien et al. (2012) [15]	-	Jan 2008 – Dec 2011	C, K, P functions, malignancy or DM, medication affecting muscle turnover	57 (63% ♂)	55.5±8.1 years, CP 8.9, MELD 13.9	RA (72%), VB (25%), both (3%)	PTFE-C	7mmHg	13.5M	-		
											BMI	+1.2 (NSD)
											SMA	+7.4 (p<0.05)
											MA	-1.1 (NSD)
											VAT	-7.6 (NSD)
											SAT	-9.7 (p<0.05)

Footnote: AF – ascitic-free, **AH** – arterial hypertension, **ASC** – ascites, **BCM** – body cell mass (kg), **BM(S)** – bare metallic (stent), **BMI** – body mass index (kg/m^2), **C** – cardiac, **CHD** – coronary heart disease, **CP** – Child-Pugh score, **DM** – diabetes mellitus, **DLM** – dry lean mass (kg), **FM** – fat mass (% of total body weight/*kg), **F10/F30** – force of m. adductor policis (%), **HCC** – hepatocellular carcinoma, **K** – kidney, **kg** – kilogram, **L** – liver, **M** – months, **MA** – muscle attenuation (Hounsfield units), **MAFA** – mid-arm fat area (cm^2), **MAMA** – mid-arm muscle area (cm^2), **MELD** – model for end-stage liver disease, **MRR** – muscle relaxation rate (m. adductor policis) (%), **mmHg** – millimeters of mercury, **NDS** – no significant difference, **OW** – overweight, **P** – pulmonary, **PTFE-C** – polytetrafluoroethylene cover, **PPG** – portal pressure gradient, **PS** – prospective, **PVT** – portal vein thrombosis, **RA** – refractory/recurrent ascites, **RH** – refractive hydrothorax, **RS** – retrospective, **SAT** – subcutaneous adipose tissue (cm^3/3 mm), **SATI** – subcutaneous adipose tissue index (cm^2/m^2), **SFA** – subcutaneous fat area (cm^2), **SFT** – subcutaneous fat thickness (cm), **SMA** – skeletal muscle area (cm^2), **SMI** – skeletal muscle index (cm^2/m^2), **SPB** – spontaneous bacterial peritonitis, **TPMA** – total psoas muscle area (mm^2), **TPMT** – transversal right psoas muscle thickness at the umbilical level/height (mm/m), **TIPS** – transjugular intrahepatic portosystemic shunt, **Tx** – liver transplantation, **UNW** – under or normal weight, **VAT** – visceral adipose tissue (cm^3/3 mm), **VATI** - visceral adipose tissue index (cm^2/m^2), **VB** – variceal bleeding, **VFA** – visceral fat area (cm^2), **W** – weight (kg), **µ** – mean, **-** – no specific information. Continuous variables are expressed as means ± standard deviations (or standard error of mean) or as medians and interquartile ranges (**IQR**).

PRISMA 2020 Checklist

Section and Topic	Item #	Checklist item	Location where item is reported
**TITLE**			
Title	1	Identify the report as a systematic review.	Title (p 1)
**ABSTRACT**			
Abstract	2	See the PRISMA 2020 for Abstracts checklist.	Abstract (p 1)
**INTRODUCTION**			
Rationale	3	Describe the rationale for the review in the context of existing knowledge.	lines 41- 48
Objectives	4	Provide an explicit statement of the objective(s) or question(s) the review addresses.	lines 48- 50
**METHODS**			
Eligibility criteria	5	Specify the inclusion and exclusion criteria for the review and how studies were grouped for the syntheses.	lines 68-75
Information sources	6	Specify all databases, registers, websites, organisations, reference lists and other sources searched or consulted to identify studies. Specify the date when each source was last searched or consulted.	lines 57-66
Search strategy	7	Present the full search strategies for all databases, registers and websites, including any filters and limits used.	lines 68-75
Selection process	8	Specify the methods used to decide whether a study met the inclusion criteria of the review, including how many reviewers screened each record and each report retrieved, whether they worked independently, and if applicable, details of automation tools used in the process.	lines 77-83
Data collection process	9	Specify the methods used to collect data from reports, including how many reviewers collected data from each report, whether they worked independently, any processes for obtaining or confirming data from study investigators, and if applicable, details of automation tools used in the process.	lines 77-83
Data items	10a	List and define all outcomes for which data were sought. Specify whether all results that were compatible with each outcome domain in each study were sought (e.g. for all measures, time points, analyses), and if not, the methods used to decide which results to collect.	lines 84-91
	10b	List and define all other variables for which data were sought (e.g. participant and intervention characteristics, funding sources). Describe any assumptions made about any missing or unclear information.	lines 84-91
Study risk of bias assessment	11	Specify the methods used to assess risk of bias in the included studies, including details of the tool(s) used, how many reviewers assessed each study and whether they worked independently, and if applicable, details of automation tools used in the process.	lines 93-96
Effect measures	12	Specify for each outcome the effect measure(s) (e.g. risk ratio, mean difference) used in the synthesis or presentation of results.	lines 143, 154, 174
Synthesis methods	13a	Describe the processes used to decide which studies were eligible for each synthesis (e.g. tabulating the study intervention characteristics and comparing against the planned groups for each synthesis (item #5)).	-
	13b	Describe any methods required to prepare the data for presentation or synthesis, such as handling of missing summary statistics, or data conversions.	lines 89 - 91
	13c	Describe any methods used to tabulate or visually display results of individual studies and syntheses.	line 93
	13d	Describe any methods used to synthesize results and provide a rationale for the choice(s). If meta-analysis was performed, describe the model(s), method(s) to identify the presence and extent of statistical heterogeneity, and software package(s) used.	no meta-analysis
	13e	Describe any methods used to explore possible causes of heterogeneity among study results (e.g. subgroup analysis, meta-regression).	no meta-analysis
	13f	Describe any sensitivity analyses conducted to assess robustness of the synthesized results.	no meta-analysis
Reporting bias	14	Describe any methods used to assess risk of bias due to missing results in a synthesis (arising from reporting biases).	line 93

PRISMA 2020 Checklist

Section and Topic	Item #	Checklist item	Location where item is reported
assessment			
Certainty assessment	15	Describe any methods used to assess certainty (or confidence) in the body of evidence for an outcome.	no meta-analysis
**RESULTS**			
Study selection	16a	Describe the results of the search and selection process, from the number of records identified in the search to the number of studies included in the review, ideally using a flow diagram.	Figure 1
	16b	Cite studies that might appear to meet the inclusion criteria, but which were excluded, and explain why they were excluded.	-
Study characteristics	17	Cite each included study and present its characteristics.	Results section
Risk of bias in studies	18	Present assessments of risk of bias for each included study.	Figure 2
Results of individual studies	19	For all outcomes, present, for each study: (a) summary statistics for each group (where appropriate) and (b) an effect estimate and its precision (e.g. confidence/credible interval), ideally using structured tables or plots.	Results section
Results of syntheses	20a	For each synthesis, briefly summarise the characteristics and risk of bias among contributing studies.	No meta-analysis
	20b	Present results of all statistical syntheses conducted. If meta-analysis was done, present for each the summary estimate and its precision (e.g. confidence/credible interval) and measures of statistical heterogeneity. If comparing groups, describe the direction of the effect.	No meta-analysis
	20c	Present results of all investigations of possible causes of heterogeneity among study results.	lines 226-290
	20d	Present results of all sensitivity analyses conducted to assess the robustness of the synthesized results.	No meta-analysis
Reporting biases	21	Present assessments of risk of bias due to missing results (arising from reporting biases) for each synthesis assessed.	Figure 2
Certainty of evidence	22	Present assessments of certainty (or confidence) in the body of evidence for each outcome assessed.	-
**DISCUSSION**			
Discussion	23a	Provide a general interpretation of the results in the context of other evidence.	Throughout Discussion
	23b	Discuss any limitations of the evidence included in the review.	Throughout Discussion
	23c	Discuss any limitations of the review processes used.	-
	23d	Discuss implications of the results for practice, policy, and future research.	Throughout Discussion
**OTHER INFORMATION**			
Registration and protocol	24a	Provide registration information for the review, including register name and registration number, or state that the review was not registered.	Not registered
	24b	Indicate where the review protocol can be accessed, or state that a protocol was not prepared.	Not prepared
	24c	Describe and explain any amendments to information provided at registration or in the protocol.	NA
Support	25	Describe sources of financial or non-financial support for the review, and the role of the funders or sponsors in the review.	lines 276-277
Competing	26	Declare any competing interests of review authors.	line 280

PRISMA 2020 Checklist

Section and Topic	Item #	Checklist item	Location where item is reported
interests			
Availability of data, code and other materials	27	Report which of the following are publicly available and where they can be found: template data collection forms; data extracted from included studies; data used for all analyses; analytic code; any other materials used in the review.	Supplementary information

From: Page MJ, McKenzie JE, Bossuyt PM, Boutron I, Hoffmann TC, Mulrow CD, et al. The PRISMA 2020 statement: an updated guideline for reporting systematic reviews. BMJ 2021;372:n71. doi: 10.1136/bmj.n71

For more information, visit: http://www.prisma-statement.org/
